# Proteomic Analysis of the Low Molecular Mass Fraction of Newly Diagnosed and Recurrent Glioblastoma CUSA Fluid: A Pilot Investigation of the Peptidomic Profile

**DOI:** 10.3390/ijms26136055

**Published:** 2025-06-24

**Authors:** Alexandra Muntiu, Federica Vincenzoni, Diana Valeria Rossetti, Andrea Urbani, Giuseppe La Rocca, Alessio Albanese, Edoardo Mazzucchi, Alessandro Olivi, Giovanni Sabatino, Claudia Desiderio

**Affiliations:** 1 Dipartimento di Scienze Biotecnologiche di Base, Cliniche Intensivologiche e Perioperatorie, Università Cattolica del Sacro Cuore, 00168 Rome, Italy; 2 Fondazione Policlinico Universitario A. Gemelli IRCCS, Catholic University, 00168 Rome, Italy; 3 Istituto di Scienze e Tecnologie Chimiche “Giulio Natta”, Consiglio Nazionale delle Ricerche, 00168 Rome, Italy; 4 Institute of Neurosurgery, Fondazione Policlinico Universitario A. Gemelli IRCCS, Catholic University, 00168 Rome, Italy; 5 IRCCS Regina Elena National Cancer Institute, 00128 Rome, Italy

**Keywords:** glioblastoma multiforme, brain tumor, CUSA fluid, peptidomics, mass spectrometry

## Abstract

Glioblastoma multiforme (GBM) is a highly aggressive, treatment-resistant grade IV brain tumor with poor prognosis that grows rapidly and invades surrounding tissues, complicating surgery and frequently recurring. Although the crucial role of endogenous peptides has been highlighted for several tumors, the specific peptidomic profile of GBM remains unexplored to date. This study aimed to perform a preliminary characterization of the low molecular mass proteome fraction of Cavitron Ultrasonic Surgical Aspirator (CUSA) fluid collected from different tumor zones, i.e., the core and tumor periphery of newly diagnosed (ND) and recurrent (R) GBM. The samples, pooled by tumor type and collection zone, were centrifuged through molecular cut-off filter devices to collect the non-retained fraction of the proteome <10 kDa for direct full-length LC-MS analysis. A total of 40 and 24 peptides, fragments of 32 and 18 proteins, were marked as ND and R GBM COREs, respectively, while 132 peptides, fragments of 46 precursor proteins, were identified as common and included proteins which were cancer-related or involved in GBM pathophysiology. Besides providing a preliminary overview of the unexplored peptidome of GBM, this pilot study confirms peptidomics as a promising tool to discover potential GBM biomarkers in the perspective of clinical applications increasingly oriented towards a precision medicine approach. Data are available via ProteomeXchange with the identifier PXD060807.

## 1. Introduction

Glioblastoma (GBM) isocitrate dehydrogenase (IDH) wild type is classified as a highly aggressive cancer with poor prognosis according to the World Health Organization (WHO) classification of CNS tumors [1,2]. For patients undergoing the standard treatment protocol, which includes surgical resection followed by radiation therapy and chemotherapy with temozolomide, the median survival is approximately 15 to 23 months and decreases with age [3,4]. The incidence of GBM ranges between two and five cases per 100,000 people annually in the United States and Europe. The highest incidence of GBM is observed in individuals aged between 75 and 84 years; after 85 years, the incidence of this tumor decreases [5]. This brain tumor represents a therapeutic challenge due to its rapid growth and tendency to relapse [3]. One approach to improve the surgical resection of GBM, approved in 2017, is the pre-surgery administration of 5-aminolevulinic acid (5-ALA) to the patient to induce intraoperative tumor tissue fluorescence [6]. Nonetheless, the total removal of the tumor remains a difficult task due to the invasive nature of GBM, thus resulting in a recurrence rate of over 90% [3]. Identifying the tumor relapse in its early stages could significantly influence the prognosis, treatment approach, and life expectancy [7]. The discovery of biomarkers and molecular targets for the development of new therapies and diagnostic applications is therefore a primary goal. In these fields of investigation, peptidomics has emerged as a promising science to identify molecular markers for predictive, preventive, and personalized medicine applications [8,9]. A recent review highlights the role of immunopeptidomics in cancer investigations, summarizing the applied methodologies as well as the results of related proteogenomic and mass spectrometry studies for the discovery of non-canonical tumor peptide antigens [10]. Special attention is also paid to the identification of peptides encoded by long non-coding RNAs and to their role in cancer [11,12].

In our previous studies [13,14], we first explored the proteomic profile of GBM tissue aspirate fluid obtained via a CUSA (Cavitron Ultrasonic Surgical Aspirator), and distinct profiles were found to mark the newly diagnosed (ND) and recurrent (R) GBMs, as well as different zones, i.e., the tumor core and periphery. In those investigations we applied a shotgun proteomic approach, analyzing protein mixtures after enzymatic digestion, an approach that makes it difficult to simultaneously distinguish and identify the naturally occurring peptidome still unexplored in GBM CUSA fluid and the object of the present investigation. We therefore here present the results of LC-MS proteomic analysis of the intact/undigested low molecular mass fraction of the CUSA fluid proteome obtained by molecular sieving on 10 kDa cut-off Filter-Aided Sample Preparation (FASP) devices. This investigation offers an interesting and yet unexplored analysis of the small proteins and peptides, including cryptides [15], and related proteolytic events occurring in vivo, featuring GBM tumors. This preliminary overview obtained on pilot CUSA fluid samples, pooled by tumor type and zone of collection, provides interesting insights for future investigations and biomarker exploration in the perspective of new clinical applications, after appropriate validation, and additionally, for a deeper comprehension of the molecular processes underlying the onset and progression of the disease. The aim of the proteomic analysis of CUSA samples collected from different tumor regions is not only to identify the distinct molecular profiles associated with them, but also to explore the potential application of this information in mass spectrometry-guided surgery, an emerging approach in the field of precision medicine. A stepwise profiling from CORE to non-fluorescent areas adjacent to healthy tissue could also allow us to evaluate tumor infiltration beyond visible margins, a crucial aspect to improve radical surgical removal while minimizing the risk of recurrence.

## 2. Results and Discussion

**Figure 1:**
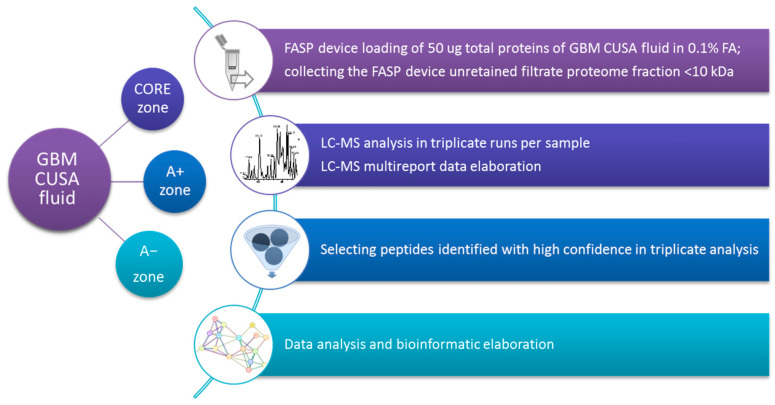
Sample processing and data analysis pipeline applied to CUSA aspirate fluid sample pools of ND and R GBMs collected from the tumor CORE zone and 5-ALA induced fluorescence positive (A+) and negative (A−) peripheral areas.

The proteomic analysis was performed on CUSA aspirate fluid of both ND and R GBM CORE zones and peritumoral areas defined by 5-ALA intraoperative-induced fluorescence, which distinguishes the fluorescent tumor tissue (CORE zone and 5-ALA positive tumor periphery, A+) from the non-fluorescent non-tumoral peripheral tissue (5-ALA negative periphery, A−), defining the tumor margins of resection. The low molecular mass fraction was obtained by filtration of the CUSA fluid through 10 kDa molecular cut-off FASP devices after dilution with formic acid aqueous solution, following our previously described C-FASP approach [16]. The fraction was analyzed by UHPLC-ESI-Orbitrap-MS after lyophilization and redissolution in a concentrated volume of formic acid aqueous solution for small proteins’ and peptides’ characterization in their intact forms. The proteomic analysis identified with high confidence a total number of 781, 605 and 713 peptides in the ND GBM CUSA CORE, A+, and A− regions, respectively (Supplementary Table S1). For the R GBM CUSA pools, the analysis identified, in the tumor CORE and A+ and A− tumor peripheries, 569, 451, and 451 peptides, respectively (Supplementary Table S2). These data were further filtered for triplicate data repeatability and unique protein groups by exclusively selecting the peptides identified with high confidence in all the three analytical replicates per sample pool, thus removing the results that did not show analytical repeatability. Figure 1 illustrates the pipeline applied to sample processing and data elaboration.

As result of this stringent data filtering, 279, 213, and 300 peptide sequences were effectively selected in the ND GBM CUSA fluid CORE, A+, and A− zones, respectively, pertaining to 82, 63, and 84 peptide precursor proteins, respectively (Supplementary Table S1). Similarly, R GBM CUSA fluid analysis resulted in the identification of 218, 168, and 166 peptide sequences originating from 68, 52, and 39 peptide precursor proteins, respectively, in the CORE, A+, and A− zones (Supplementary Table S2).

The peptidomic data obtained were analyzed and compared, considering either the peptide sequences identified or the relative proteins of origin, to investigate specific classifications and their role in GBM by cross-examining the specific database and the literature data, and to explore the molecular differences between the tumor types and zones of collection. Indeed, a first screening of the proteomic data obtained was performed on peptide fragments’ precursor proteins to preliminary explore exclusive and common elements of ND and R GBM tumor types and of the relative zones of collection, including the tumor zone (CORE and A+ fluorescent periphery) and the peritumoral tissue (not fluorescent A− periphery).

**Figure 2:**
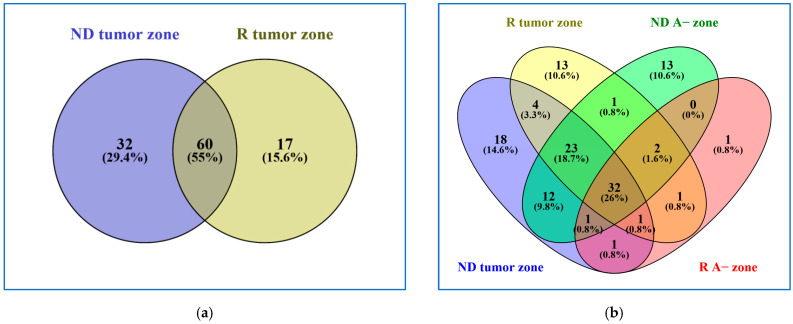
Venn diagrams resulting from grouping analysis of (**a**) ND versus R GBM tumor zones and (**b**) ND and R GBM tumor zones versus the surrounding peripheral tissue (A− zone).

Another analysis was then dedicated to the specific comparison of only ND and R GBM tumor zones, including both the CORE and A+ zones of each tumor type (Figure 2a). ND and R GBM tumor zones showed distinct profiles, as demonstrated from the identification of 32 and 17 exclusive precursor proteins, respectively. The ND GBM tumor zone shared with the tumor relapse zone 55% of the precursor proteins identified, protein elements which can be considered to represent the central molecular profile of the GBM tumor, although its exclusiveness with respect to the normal A− zone needed to be investigated. Therefore, the grouping analysis of the ND and R GBM tumor zones was also extended to the data of the relative A− zone (Figure 2b).

At first glance, most of the peptide precursor proteins identified in the A− zone were also identified in the relative tumor zone. This was particularly evident for the R GBM A− data, showing only one precursor protein not in common with the tumor zone. Special attention was therefore paid to the classification of the peptide protein precursors commonly identified in the A− and tumor zones, in relation to cancer and GBM disease, following the database of reference as well as the literature data. Peptide precursor proteins common to all zones could indeed represent either elements featuring normal tissue or proteins which are cancer-related and involved in GBM disease onset and progression, a difficult task to investigate since there is a lack of control brain tissue as a reference.

This preliminary data exploration highlighted the main differences between ND and R GBMs as residing in the CORE zone, which was therefore the starting point of comparative analysis of the peptidomic data, as illustrated in the following paragraphs, underlining the most relevant results.

### 2.1. Comparative Analysis of the ND and R GBM Tumor CORE Zones

Table 1 lists the amino acid sequence, experimental monoisotopic mass (*m*/*z*, MH+), PTMs, and precursor protein sequence position of the 40 peptides exclusively identified in the ND GBM CUSA CORE with respect to the R GBM CORE; nonetheless, it is worthy of mention that some of these peptides were also identified in the A+ and/or A− zones of the ND GBM tumor. In the table, the peptide protein precursors’ gene names and Uniprot accession numbers are also reported. The identified peptides are ordered according to the zone of identification, with peptides exclusively identified in the ND CORE reported first, followed by the peptides also identified in the A+ and/or the A− ND zones. In Supplementary File S1, a different visualization of the identified peptides in Table 1, grouped differently by precursor protein of origin, is reported. According to the Human Protein Atlas (HPA) database (https://www.proteinatlas.org, accessed on 11 June 2024), it is noteworthy that some of these peptides are fragments of products of genes “highly expressed in GBM”, such as the Alpha-crystallin B chain (CRYAB), astrocytic phosphoprotein PEA-15 (PEA15), Osteopontin (SPP1), and Neuromodulin (GAP43), or “associated with poor prognosis in GBM”, like Sarcosine dehydrogenase, mitochondrial (SARDH), or classified as “cancer-related”, such as the UV excision repair protein RAD23 homolog B (RD23B), Apolipoprotein A-II (APOA2), Fructose-bisphosphate aldolase A (ALDOA), Triosephosphate isomerase (TPIS), Albumin (ALB), and CD44 antigen (CD44). These peptides are specifically underlined in Table 1. A significant number of the identified peptides in Table 1 are N- or C-terminal sequence traits of the relative precursor protein, parts of the sequence probably more accessible to proteolytic cleavage, and some of them carry N-terminal acetylation or methionine oxidation PTMs. It is worthy of mention that two precursor proteins in Table 1, namely the CD44 antigen and Osteopontin (Uniprot accessions P16070 and P10451, respectively), have been identified as exclusive of the GBM ND CUSA CORE also by the bottom-up approach, as resulting from our previous investigation [13].

In an attempt to explore whether there are known proteases to cleave the substrates at the specific sequences to produce the peptide fragments identified in Table 1, we used the freely available MEROPS Peptidase Database (https://www.ebi.ac.uk/merops, accessed on 12 September 2024) [17].

The peptide fragments 161–175 (C-terminal, 1638.953 *m*/*z*, MH^+^) and 24–32 (1139.518 *m*/*z*, MH^+^) of Alpha-crystallin B chain (CRYAB) could potentially be generated by the proteolytic cleavage of Matrix metalloproteinase-9 (MMP9). Interestingly, MMP9 is reported as up-regulated in glioma tissues, with its expression correlating with the tumor grade [18,19,20,21]. Another substrate of MMP9 is Osteopontin (SPP1) [22], a protein present in several tissues and organs, including the central nervous system [23]. MMP9 cleaves Osteopontin at a specific site between residues 301 and 302, generating the C-terminal fragment NER3 (1387.638 *m*/*z*, MH^+^) [24], a fragment that is present in Table 1. This peptide, which is neurotoxic and pro-inflammatory, was reported as a potential diagnostic biomarker for amyotrophic lateral sclerosis (ALS) [24]. It is noteworthy that tumor invasion is facilitated by the remodeling of the extracellular matrix via MMP expression [25], but also by the action of GAP43, a nerve growth-associated protein of which we interestingly identified two C-terminal peptide fragments, 219–238 and 220–238 (2283.961 and 2154.921 *m*/*z*, MH^+^, respectively). Although the function of these peptide fragments is unknown, the GAP43 protein plays a role in the formation of multicellular network structures delivering microtube-dependent tumor cells’ interconnection and invasion, proliferation, and radio-resistance features [26,27]. GAP43 was reported to mediate the transfer of mitochondria from astrocytes to GBM cells, thus favoring tumor growth by modulating metabolic processes and signaling pathways [27].

According to the HPA database, Sarcosine dehydrogenase, mitochondrial (SARDH) is an unfavorable prognostic gene in glioma. We identified its 31 amino acid-length 863–893 peptide fragment (molecular mass 3402.681 *m*/*z*, MH^+^) carrying an oxidation PTM at M_31_. SARDH is a crucial enzyme in sarcosine metabolism, with significant implications in human health, and recent studies have highlighted its role as a biomarker in the early diagnosis of prostate cancer [28]. To the best of our knowledge, no data on the biological activity of specific protein fragments are available.

The astrocytic phosphoprotein PEA-15 is a GBM predictive marker [29], and its gene is highly expressed in this tumor according to the HPA database. In this study, we identified its acetylated N-terminal fragment 2–14 (molecular mass of 1493.716 *m*/*z*, MH^+^). This protein is known to protect glioma cells from cell death induced by the Tumor necrosis factor-related apoptosis-inducing ligand [30,31]. In addition, PEA15 regulates the expression of the CAR receptor, essential for tumor cell proliferation and migration, as well as for treatment resistance [29].

As shown in Table 1, several peptide fragments identified in the ND CUSA CORE have been also identified in the ND GBM tumor periphery, and particularly, the fragments 220–238 of GAP43, 90–100 of APOA2, and the fragments of SPP1, ALDOA, and CD44 are precursor proteins which are all cancer-related or previously associated to GBM disease, suggesting that, at the peptide level, the pathological process could also affect the A− area adjacent to the tumor A+ periphery, in accordance with previous indications [13].

**Figure 3:**
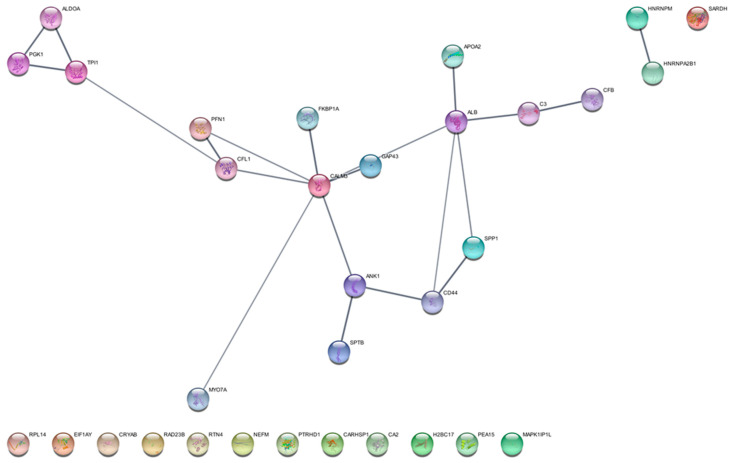
Protein functional networks resulting from Cytoscape (version 3.10.3) visualization of STRING (version 12.0) analysis (setting the high confidence 0.700 minimum required interaction score) of the peptides’ precursor proteins exclusively identified in the ND GBM CUSA CORE with respect to the R GBM CUSA CORE (list in Table 1).

**Figure 4:**
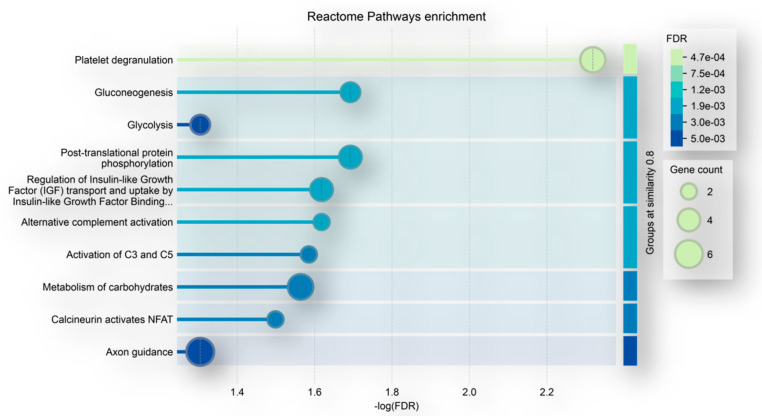
Top ten pathways, namely, Platelet degranulation, Gluconeogenesis, Glycolysis, Post-translational protein phosphorylation, Regulation of Insulin-like Growth Factor (IGF) transport and uptake by Insulin-like Growth Factor Binding Proteins (IGFBPs), Alternative complement activation, Activation of C3 and C5, metabolism of carbohydrates, Calineurin activates NFAT and Axon guidance, determined to be statistically significantly enriched by pathways enrichment analysis of the peptides’ precursor proteins exclusively identified in the ND GBM CUSA CORE with respect to the R GBM CUSA CORE (list in Table 1) (network in Figure 3).

Focusing on the precursor proteins listed in Table 1, they showed functional predicted relationships as resulting from analysis by the STRING tool (Figure 3), with the “extracellular exosomes”, including 16 elements out of the 32 totally analyzed, resulting in the “cellular components” having the highest statistically significant value of enrichment in the network (FDR value 6.46 × 10^−5^). Figure 4 depicts the list of the top 10 pathways most significantly over-represented in the network following enrichment analysis. All these pathways have been reported as involved in GBM onset and progression [32,33,34,35,36,37,38,39].

Particularly interesting is the identification of the CRYAB protein in saliva as exclusive of ND GBM in our recent investigation [40]. Therefore, CRYAB, both in its full sequence form or as selected peptide fragments, is a protein element to be deeply investigated as a GBM potential disease biomarker. Although this data match is intriguing, it has to be taken into account that the identified peptide fragments have to be considered single entities not necessarily correlated to the presence and activity of the protein of origin, and perhaps exhibiting a proper biological function inside the cell and in the extracellular environment.

**Figure 5:**
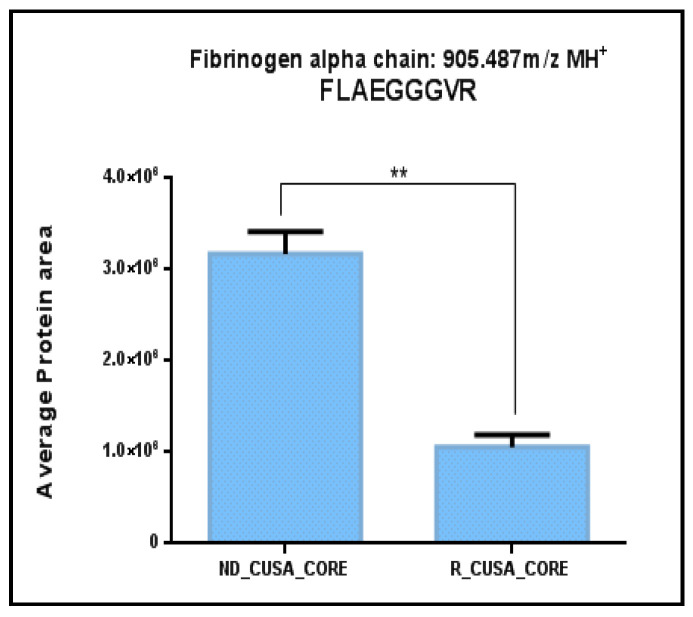
Bar chart of the label-free relative quantitation of FGA peptide fragment 27–35 in the ND and R GBM CUSA CORE pools. The area values are the mean values (± SD) of LC-MS triplicate analysis. Significant difference has been determined by *t*-test (**, *p* value *p* ≤ 0.01: *p* value: 0.0012).

A total of 132 peptides belonging to 46 precursor proteins (see Table 2) were instead identified as common to the ND and R CORE CUSA fluid pools. In Supplementary File S1, the identified peptides are presented in a different visualization, ordered based on the precursor protein of origin. These peptides, since identified in both ND and R tumor CORE specimens, can be considered to represent the central peptidome of a GBM tumor. Out of them, only five peptides were unique to the CORE zone, namely, the N-terminal fragment 2–13 of the Elongation Factor 1-Alpha 1 (EEF1A1) (2496.497 *m*/*z*, MH^+^), the fragment 27–35 of Fibrinogen Alpha Chain (FGA) (905.487 *m*/*z*, MH^+^), the fragment 406–423 of Glial Fibrillary Acidic Protein (GFAP) (2115.164 *m*/*z*, MH^+^), the acetylated N-terminal fragment 2–19 of Pyruvate kinase PKM (PKM) (2022.009 *m*/*z*, MH^+^), and the C-terminal fragment 26–44 of Thymosin beta-4 (TMSB4X) (2113.084 *m*/*z*, MH^+^), while the others showed a variable distribution between all tumor zones and the surrounding A− periphery. The fragment 27–35 of FGA was of particular interest because it was a unique peptide showing a statistically significant quantitative difference between the ND and R GBM CUSA CORE zones, as resulting from *t*-test analysis (Figure 5). This peptide is the C-terminal trait of Fibrinopeptide A, which is in turn the peptide fragment 20–35 (1536.695 *m*/*z*, MH^+^) of FGA, with a key role in the coagulation process. In the present investigation, we also identified other fragments of FGA, including the entire Fibrinopeptide A and its N-terminal-truncated form (FGA fragment 21–35, 1465.658 *m*/*z*, MH^+^), which were identified in all zones.

Fibrinopeptide A and its N-terminal-truncated form have been both identified in pediatric brain tumor tissues [41] and in the intracystic fluid of pilocytic astrocytoma pediatric brain tumors [42] in our previous investigations. Interestingly, the N-terminal-truncated form of Fibrinopeptide A is included in the SPENCER database (https://spencer.renlab.org, accessed on 15 October 2024) (peptide ID SPENP016947), which catalogues small peptides encoded by ncRNA in cancer patients [43].

Some peptides in Table 2 are fragments of expression products of genes highly expressed in GBM, according to the HPA database, such as the Hemoglobin subunit alpha (HBA1), Glial Fibrillary Acidic Protein (GFAP), Dihydropyrimidinase-related protein 2 (DPYSL2), Alpha-internexin (INA), Hemoglobin subunit beta (HBB), Microtubule-associated protein 2 (MAP2), Myelin basic protein (MBP), Aquaporin-4 (AQP4), and Protein NDRG2 (NDRG2). The presence of peptide fragments of these precursor proteins suggests their potential relevance as peptide biomarkers for GBM and underscores their possible biological activity in cancer pathways. Other peptides identified are instead fragments of proteins classified as cancer-related in the HPA database, including Transthyretin (TTR), Fibrinogen Alpha Chain (FGA), Pyruvate kinase PKM (PKM), Macrophage Migration Inhibitory Factor (MIF), Annexin A1 (ANXA1), Stathmin (STMN1), UV excision repair protein RAD23 homolog A (RAD23A), Apolipoprotein C-III (APOC3), Elongation Factor 1-Alpha 1 (EEF1A1), Fibrinogen Beta Chain (FGB), Apolipoprotein A-I (APOA1), and Protein S100-A4 (S100A4).

**Figure 6:**
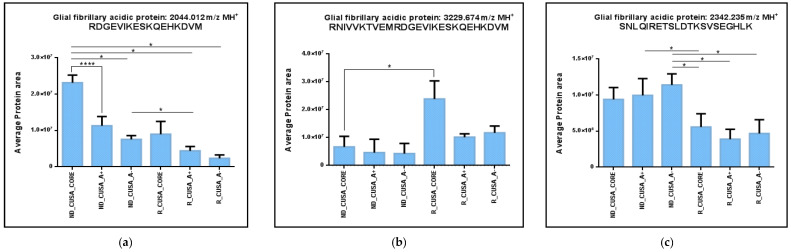
Bar chart of the label-free relative quantitation of Glial Fibrillary Acidic Protein (GFAP) precursor protein peptides (**a**) RDGEVIKESKQEHKDVM (2044.012 *m*/*z*, MH^+^), (**b**) RNIVVKTVEMRDGEVIKESKQEHKDVM (3229.674 *m*/*z*, MH+) and (**c**) SNLQIRETSLDTKSVSEGHLK (2342.235 *m*/*z*, MH^+^), showing statistically significant differences between the zones. Area values (mean value of replicate LC-MS analysis ± SD) in all samples analyzed in the GBM (ND_CUSA_CORE, A+, and A− and R_CUSA_CORE, A+, and A−) of identified shared elements (Table 2). Significant differences have been determined by one-way ANOVA and Tukey’s post-hoc test (* *p*-value < 0.05, **** *p*-value < 0.0001).

The precursor protein common to the ND and R GBM CORE zones showing the highest number of fragments identified is the Glial Fibrillary Acidic Protein (GFAP). In this study, sixteen different peptide fragments of GFAP were identified. GFAP is a well-established intermediate filament protein commonly used to identify neoplasms of glial origin, including astrocytomas and glioblastomas [44,45]. GFAP plays a crucial role in cytoskeletal remodeling and is implicated in GBM progression [45]. These fragments were differently distributed between the tumor zones. Particularly, seven GFAP peptides were identified in all zones, and one peptide (2115.164 *m*/*z*, MH^+^) was exclusive of the tumor CORE, as already discussed above. The seven ubiquitous GFAP fragments were analyzed for relative quantitation by comparing the area values in each zone, and only three peptides out of them showed statistically significant variations (Figure 6a–c).

These data are, however, difficult to interpret, because different trends for these peptides were observed, considering their distribution levels between the different zones and tumor types. The area of the GFAP C-terminal peptide oxidized at M17 (2044.012 *m*/*z*, MH^+^) showed a gradual decrease from the tumor CORE to the periphery in both the ND and R GBMs (Figure 6a). The higher levels observed in the CORE zones could reveal a potential role of this peptide in marking a GBM tumor. Differently, the GFAP C-terminal peptide 406–432 (3229.674 *m*/*z*, MH^+^) showed statistically significant higher levels in the R GBM with respect to the ND GBM, comparing the CORE zones. This peptide fragment seems, therefore, to be more represented in the tumor relapse. The GFAP fragment 375–405 (molecular mass 2342.235 *m*/*z*, MH^+^) generally showed about 10× higher levels in ND GBM CUSA samples with respect to the R GBM (Figure 6c). Interestingly, this peptide, according to Marcu et al. [46], is a tumor-associated antigen (TAA) peptide exclusive of GBM malignancy; therefore, its finding in CUSA sample is very relevant.

Other interesting GFAP peptides have been identified in the present study. In particular, the peptide with a mass of 2028.076 *m*/*z* MH^+^, detected in the ND and R GBM CORE and R GBM A+ and A− zones, has been previously identified in pediatric brain tumors [40]. Moreover, some GFAP fragments were found either unchanged or in mono- or di-oxidized forms at methionine residues, i.e., the C-terminal fragment MRDGEVIKESKQEHKDVM (unchanged, mono-, and di-oxidized forms, 2159.061, 2175.054, and 2191.046 *m*/*z*, MH^+^, respectively) and the fragment peptide RNIVVKTVEMRDGEVIKE (unchanged and mono-oxidized, 2115.164 and 2131.157 *m*/*z* MH^+^, respectively). Oxidative stress is often associated with increased tumor aggressiveness and treatment resistance [37,47]; therefore, the identification of oxidized methionine residues in different GFAP fragments could provide insights into the redox status of the tumor microenvironment. However, the possible oxidation induced during sample processing and MS analysis has to be taken into account.

Remaining in the family of intermediate filament proteins, several peptide fragments identified belong to Vimentin (VIM), playing a crucial role in the progression and aggressiveness of GBM [48]. Research studies highlight VIM involvement in various biological processes, particularly in epithelial–mesenchymal transition (EMT), essential for cancer cell invasion and metastasis [49]. Eight naturally occurring peptide fragments of VIM have been identified as common in ND and R GBM CUSA CORE samples, with three out of them corresponding to C-terminal fragments. Following the MEROPS database, the protease responsible for generating the VIM C-terminal peptides 446–466, 444–466, and 443–466 could likely be cathepsin S (CTSS), and this could be consistent with the high expression of CTSS reported in GBM [50]. The peptide 2423.117 *m*/*z* MH^+^ has been identified in ND GBM CORE and peripheral regions and in the R GBM CORE zone. Interestingly, this peptide has been identified in medulloblastoma DAOY cells in our previous investigation [51], in traumatic brain injury [52], and in blood serum samples of ovarian cancer, as catalogued in the SPENCER database. The two VIM peptides with masses of 2664.295 and 2777.378 *m*/*z* MH^+^, identified in almost all zones, have been previously identified in pediatric brain tumor tissues [42], strengthening the hypothesis of their correlation with brain tumors.

Numerous peptide fragments in Table 2 belong to Myelin basic protein (MBP), highly expressed in GBM and a diagnostic marker of brain damage [53,54]. The presence of MBP in the cerebrospinal fluid of brain tumor patients has been reported as a potential indicator of tumor development and progression [54]. In the present investigation, we identified ten different fragments of MBP common to ND and R GBM CORE zones. The MBP peptide with molecular mass 1046.550 *m*/*z* MH^+^ was also identified in the other zones. This peptide, with the sequence DTGILDSIGR, is catalogued in the SPENCER database as a “tumor-specific peptide” experimentally validated in skin cancer (peptide ID SPENP018934) and lung cancer (peptide ID SPENP025606) [55]. The gene associated to this ncRNA peptide is NONHSAF024214.2 or Inc-MBP-13.

The SPENCER database also catalogues the peptide AQAAAPASVPAQAPKR (1377.753 *m*/*z*, MH^+^) fragment of the Large ribosomal subunit protein eL29 (RPL29) protein as a “tumor-specific peptide”, validated in squamous cell carcinoma (peptide ID SPENP019510), distal cholangiocarcinoma (peptide ID SPENP013489), and acute leukemia (peptide ID SPENP001256) [55,56,57]. The associated gene name of this ncRNA peptide is NONHSAG035155.2. This peptide was detected in both the ND and R CUSA CORE zones, and in the ND tumor periphery. Both these tumor-specific fragment peptides of MBP and RPL29 could hold significant potential as novel neoantigens for cancer immunotherapy.

Of note is the identification of the C-terminal peptide fragment 312–323 of Aquaporin-4 (AQP4) with the sequence GKDQSGEVLSSV (1205.604 *m*/*z*, MH^+^), identified in all sample analyzed. According to Marcu et al. [46], this peptide is recognized as a tumor-associated antigen (TAA) specific to glioblastoma malignancy. This observation suggests a potential role of this peptide in marking the disease, as well as the progression of the disease. AQP4 is an essential water channel protein in the CNS, predominantly found in astrocytes, and crucial for glioma development and cell migration, although the relationship between its expression levels and malignancy remains a topic of debate [58]. According to the HPA database, AQP4 gene expression is significantly elevated in gliomas.

Four precursor proteins, namely PARK7, APEX1, SLC1A3, and CD99, always identified as common between the ND and R GBM CORE zones, were not included in Table 2 because the related peptide fragments identified in the two tumor types were found to be different due to the presence of different post-translational modifications. For example, for PARK7, we identified a peptide fragment carrying N-terminal acetylation in the ND GBM and both N-terminal acetylation and M_16_ oxidation in the R GBM, resulting in two different peptide entities which could have diverse biological activities or mark different processes, an interesting finding to underline, although, as previously stated, it should be noted that oxidation of methionine may occur during sample processing and MS analysis.

It is noteworthy to underline Histone H1.4 (H1-4), which was identified as an exclusive protein common to ND and R GBM CUSA fluids by the bottom-up proteomic approach in our previous investigation [13].

A tumor relapse can exhibit a different protein and peptide profile with respect to a newly diagnosed tumor, due to the occurrence of mutations and/or of different post-translational modifications [59,60]. In the present investigation, 24 peptides from 18 precursor proteins have been found to mark the R GBM CUSA CORE with respect to the ND CUSA CORE (table in Supplementary File S1, the peptides in table are listed based on the precursor protein of origin). Some of these peptides are fragments of products of genes highly expressed in GBM, such as Neurogranin (NRGN), Synapsin-1 (SYN1), Synapsin-2 (SYN2), and Nestin (NES). Others peptides are instead fragments of precursor proteins classified as cancer-related, such as the Tumor protein D52 (TPD52), 10 kDa heat shock protein, mitochondrial (HSPE1), Peroxiredoxin-2 (PRDX2), Histone H4 (H4C1), and High mobility group protein HMG-I/HMG-Y (HMGA1). These peptides are underlined in Table 3. Most of the peptides in Table 3 were exclusively identified in the R GBM CORE zone, and only a few were indeed identified in the R GBM tumor periphery.

Five peptides exclusive of the R GBM CORE, were derived from the C-terminal region of Neurogranin (NRGN), namely the NRGN peptide fragments 55–75, 57–75, 59–78, 53–78, and 59–75, as seen in Table 3. NRGN is largely expressed in the brain, where it is a key postsynaptic protein regulating calmodulin availability in the absence of calcium (Ca^2+^) [61]. Recent studies have highlighted that the C-terminal trait of NRGN undergoes enzymatic cleavage, generating fragment peptides modulated under neurodegeneration [62,63,64].

Peptide fragments of Synapsin-1 and -2 (SYN1 and 2) were exclusively identified in the R GBM CORE. In GBM, SYN1 and SYN2 proteins have been observed to be involved in synaptic vesicle cycling and regulation of neurotransmitter release at the synapse [65,66], and to interact with other genes in the molecular networks which drive tumor progression [67]. The finding of SYN1 and SYN2 peptide fragments in the R GBM CUSA CORE is therefore interesting, although the specific functions of these peptides have never been outlined, to the best of our knowledge.

A short peptide of 1246.595 *m*/*z* (MH^+^) of the Nestin protein (NES) was also identified. The NES gene is highly expressed in gliomas, according to the HPA, and it was recognized as a crucial marker in studies on GBM tumor cells [68,69,70]. NES is associated with cellular proliferation, stem-like features, and therapeutic resistance [70,71]. Its expression is particularly elevated in recurrent gliomas [67,70], significantly contributing to the severity of the disease and the poor survival observed in patients [70,72]. Therefore, the identification of its peptide fragment in the R GBM CORE is consistent with previous findings, suggesting a potential role of this peptide, exclusively identified in the tumor relapse, to be further investigated. Of note, the NES and Heterogeneous nuclear ribonucleoprotein U (HNRNPU) precursor proteins of Table 3 have been also identified as exclusive of the R GBM CORE zone by the bottom-up proteomic approach in our previous investigation [13].

Exclusive of the R GBM CORE area was the identification of the C-terminal peptide of the cancer-related protein High mobility group A1 (HMGA1) (https://www.proteinatlas.org, accessed on 11 June 2024) [73]. HMGA1 overexpression is associated to several cancers [74,75,76,77], including recurrent GBM, and correlated to poor prognosis and increased tumor invasiveness; therefore. the finding of its peptide fragment 89–107 in the R GBM CUSA CORE may be consistent. The peptide corresponds to the acidic C-terminal tail of HMGA1 and includes the last amino acid of the AT hook 3 chromatin unfolding domain of the protein (corresponding to the sequence trait 78–89) [78]. The C-terminal peptide contains serine residues which can undergo phosphorylation PTM by casein kinase II [79]. The HMGA1 C-terminal peptide has been previously identified in ascites samples of ovarian cancer [80], and it is catalogued in the SPENCER database (Peptide ID: SPENP011841), suggesting a potential role for this peptide in the disease to be further investigated. It is furthermore noteworthy that HMGA1 interaction with VIM promotes cell migration and tumor invasiveness and facilitates tumor recurrences, contributing to the poor prognosis of GBM [81], and, interestingly, VIM was found as commonly expressed in the ND and R GBM CUSA CORE zones.

### 2.2. Analysis of the GBM CUSA A+ Zone: The ND and R GBM Exclusive and Shared Peptides

The analysis of the common and exclusive peptidomic profiles of the ND and R GBM CORE zones places particular attention on some peptides of Table 1, Table 2 and Table 3 identified in both the CORE and the A+ zones, suggesting the presence of a conserved set of peptides in these two regions. These peptides could be of help to more accurately define the margins of resection of the tumor area through, e.g., the challenging technique of mass spectrometry-guided surgery.

Table 1 lists six peptide sequences identified in both the CUSA CORE and the A+ zones of the ND GBM, namely, the N-terminal fragment 2–14 of PEA15 (1493.716 *m*/*z*, MH^+^), the C-terminal fragment 242–249 of TPI1 (900.518 *m*/*z*, MH^+^), the C-terminal fragment 404–417 of PGK1 (1411.782 *m*/*z*, MH^+^), the fragments 423–432 and 432–447 of ALB (1182.618 and 1830.091 *m*/*z*, MH^+^), and the fragment 16–28 of PTRHD1 (1374.695 *m*/*z*, MH^+^). Similarly, Table 3 shows six sequences shared between the CORE and the A+ zones of R GBM, namely, the C-terminal fragment 89–107 of HMGA1 (2237.003 *m*/*z*, MH^+^), the N-terminal 2–13 of GRINA (1361.637 *m*/*z*, MH^+^), the fragment 57–75 of NRGN (1405.702 *m*/*z*, MH^+^), the fragment 3–13 of TUBB (1154.566 *m*/*z*, MH^+^), the fragment 655–665 of HNRNPU (1314.729 *m*/*z*, MH^+^) and the fragment 110–122 of RPS17 (1405.697 *m*/*z*, MH^+^). These data could suggest common molecular pathways or conserved activities of specific proteases in both the CORE and the A+ zones. Finally, Table 2 highlights two sequences commonly identified in the CORE and A+ zones of both ND and R GBMs, namely, the fragment 275–291 of GAPDH (1922.819 *m*/*z*, MH^+^) and the N-terminal fragment 2–27 of TMSB10 (2980.501 *m*/*z*, MH^+^), which therefore seem to define the tumor tissue independently from the tumor type.

Interestingly, 13 peptides were instead exclusively identified in the A+ zone (Table 4). Their relative precursor proteins were Tubulin alpha-1C chain (TUBA1C), Apolipoprotein E (APOE), Septin-8 (SEPTIN8), Heterochromatin protein 1-binding protein 3 (HP1BP3), and High mobility group nucleosome-binding domain-containing protein (HMGN4) for the ND GBM, and Histone H3.1 (H3C1) and Mitochondrial ribosome-associated GTPase 2 (MTG2) for the R GBM (Table 4).

**Figure 7:**
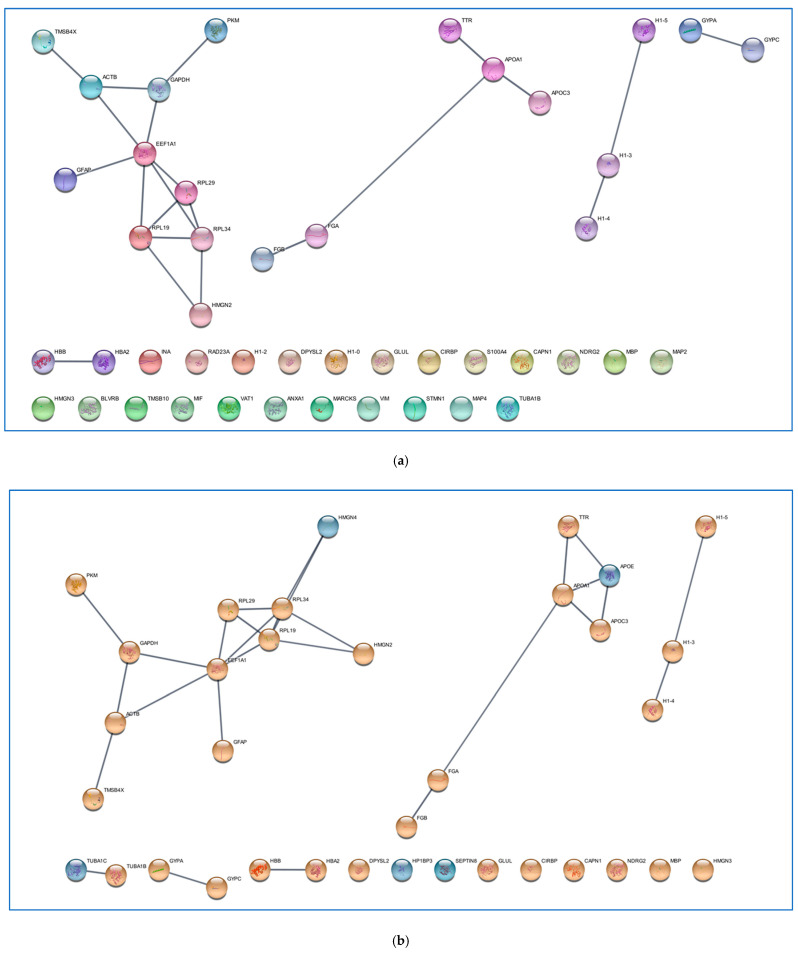

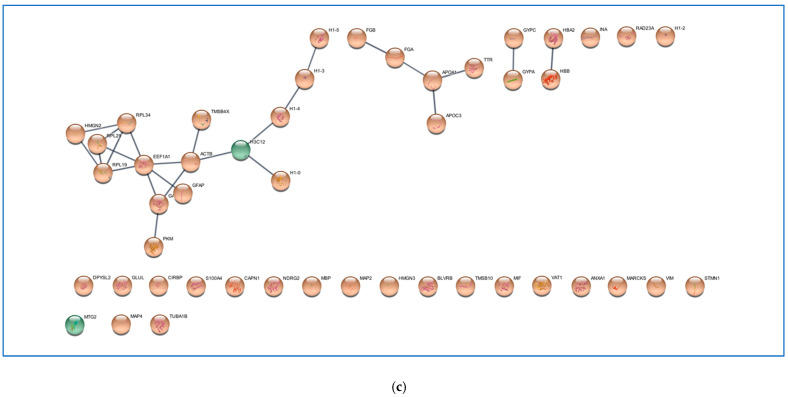
Protein functional networks resulting from Cytoscape (version 3.10.3) visualization of STRING (version 12.0) analysis (setting the high confidence 0.900 minimum required interaction score) of the peptides’ precursor proteins common to the ND and R GBM CUSA CORE zones (list in Table 2) alone (panel (**a**)), and in the presence of the peptides’ precursor proteins exclusive of the ND GBM A+ zone (blue nodes) (list in Table 4) (panel (**b**)), and exclusive of the R GBM A+ zone (green nodes) (list in Table 4) (panel (**c**)). The protein nodes common to ND and R GBM CUSA CORE zones in panel a, are single-colored in panels b and c for better differentiation.2.3. The Exclusive Peptides of the A− Zone.

It was then interesting to verify potential relationships between the peptides’ precursor proteins exclusive of the A+ zone in Table 4, with the precursor proteins common to the ND and R GBM CORE zones of Table 2, by the STRING tool (Figure 7). The results revealed that APOE and HMGN4, exclusive peptide precursor proteins of the ND GBM A+ zone, showed predicted relationships with the two main clusters of interaction formed by the common proteins (Figure 7a,b); therefore, the profile of the A+ zone seems to be correlated to the GBM CORE profile. In the brain, APOE undergoes enzymatic fragmentation, and different fragmentation patterns have been studied in relation to Alzheimer’s disease [82]. The finding of this unique APOE fragment in GBM CUSA fluid, and specifically marking the A+ zone, is intriguing, since specific traits of the protein have been associated to different biological activities [82]. H3C1, on the other hand, not only forms interactions with the main clusters of the network in Figure 7a but also serves as a bridge between the different axes (Figure 7c), resulting in a central position in the network. Alterations of H3C1 variants were associated with glioma malignancy [83]. The histone has been also identified in pediatric brain tumors, such as diffuse intrinsic pontine glioma (DIPG) [84].

The data of the identified peptides are in Table 5. Peptide fragments of 13 proteins out of the total 14 listed in Table 5, namely, Vesicle-associated membrane protein 3 (VAMP3), Spectrin alpha chain, erythrocytic 1 (SPTA1), Vitronectin (VNT), Excitatory amino acid transporter 2 (SLC1A2), Pyruvate kinase PKLR (PKLR), Hemoglobin subunit delta (HBD), Microtubule-associated protein tau (MAPT), Eukaryotic translation initiation factor 4H (EIF4H), Band 3 anion transport protein (SLC4A1), Tubulin beta-2A chain (TUBB2A), Small ribosomal subunit protein uS11 (RPS14), Actin, cytoplasmic 2 (ACTG1), and Cytochrome c oxidase subunit 6B1 (COX6B1), resulted in the characterization of the A− zone of the ND GBM. The R GBM A− zone was instead marked by fragments of only one precursor protein, namely Histone H3.3C (H3-5).

**Figure 8:**
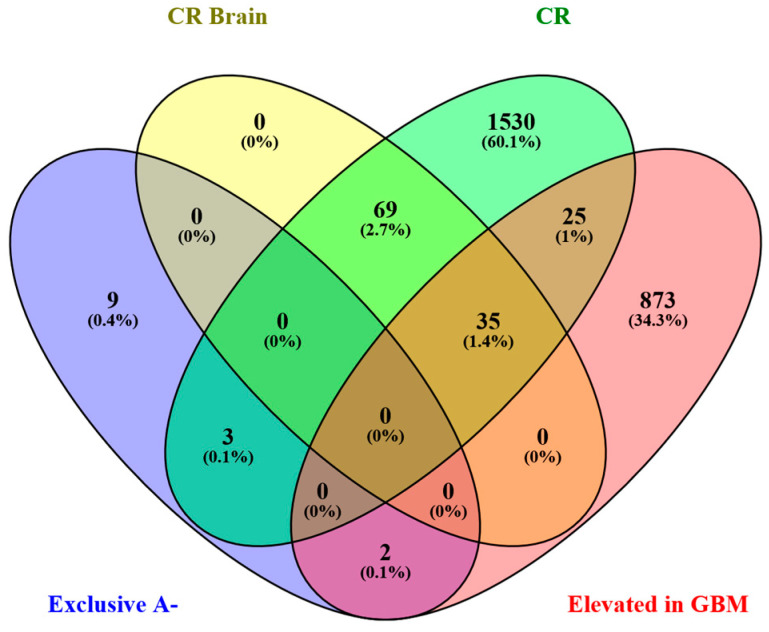
Venn diagram showing the distribution of the precursor proteins exclusive of the A− zone between the proteins classified as cancer-related (CR), tissue-elevated cancer-related genes in the brain (CR Brain), and genes elevated in glioblastoma multiforme (Elevated in GBM), based on the HPA database of reference.

These peptides could define the normal brain tissue surrounding the tumor peripheral area, as well as, conversely, represent biomolecules with a role in tumor dissemination, thus infiltrating the brain tissue. Interestingly, resulting from grouping analysis of the precursor proteins in reference to specific classifications in the HPA database (Figure 8), Table 5 showed the identification of peptide fragments of cancer-related proteins in the A− area, namely of the VNT, VAMP3, and EIF4H proteins, which could play a role in tumor invasion and relapse if mirroring the activity of the parent proteins. Furthermore, SLC1A2 and TUBB2A, although not classified as cancer related, were classified as products of genes with elevated expression in GBM. Their finding in the A− tumor periphery was therefore unexpected. On the other hand, because these two proteins are also classified as “elevated in brain”, their identification in the A− zone is difficult to interpret. Finally, the peptide fragments of nine precursor proteins (SPTA1, PKLR, HBD, MAPT, RPS14, ACTG1, COX6B1, SLC4A1, and H3-5), not included in these specific classifications as tumor-related, could define, through the presence of their specific fragments, the margins of normal brain tissue. The unavailability of control brain tissue makes the interpretation of the profile of the A− zone difficult. However, the peculiar peptidomic profiles of the diverse tumor zones analyzed make the obtained results intriguing for further investigations.

## 3. Materials and Methods

### 3.1. Chemicals

5-aminolevulinic acid (5-ALA) was from Medac (Wedel, Germany). Formic acid (FA), water, acetonitrile (ACN), and Filter-Aided Sample Preparation (FASP) centrifugal filter units Microcon 10 were from Merck (Darmstadt, Germany). All organic solvents were of LC-MS grade. Formic acid (FA) (≥99%, for LC-MS) was from VWR Chemicals (VWR International s.r.l., Milan, Italy). Protease inhibitor cocktail (PIC) (AEBSF, aprotinin, bestatin, E-64, EDTA, and leupeptin) was from Sigma-Aldrich (St. Louis, MO, USA).

### 3.2. Sample Collection and Pretreatment

CUSA fluid samples were collected from seven patients (four ND, median age 64.5 ± 11.7 years; three R, median age 48.7 ± 8.1 years) affected by a GBM IDH-1 wild type brain tumor under informed consent and approval of the Ethical Committee of the Catholic University of Sacred Heart—Fondazione Policlinico Gemelli in Rome (reference number 13891/18 ID 2015, Prot. N. 0020786/18, 18 May 2018) (patient data in Supplementary Table S3). CUSA fluid samples were collected and pretreated as previously described [13]. Briefly, CUSA fluid samples were thawed at room temperature, centrifuged at 1200 rpm, 4 °C for 5 min to precipitate cells and solid material, and pooled based on the GBM state (ND vs. R) and tumor region of collection (tumor core, CUSA CORE; 5-ALA positive periphery, CUSA A+; and 5-ALA negative periphery, CUSA A−), and the total protein content measured by Bradford assay. A volume of each CUSA fluid pool corresponding to 50 µg of total protein content was diluted with 0.1% (*v*/*v*) aqueous FA solution up to a final volume of 200 µL, transferred to the FASP filter device with 10 kDa molecular cut-off and centrifuged at 14,000 rpm for 15 min at 4 °C, after conditioning the filter, following the optimized C-FASP protocol [16]. The fraction unretained by the filter, i.e., the low molecular mass proteome fraction <10 kDa, was collected for proteomic profiling in the intact form, added with the protease inhibitor cocktail at a ratio of 1:20 (*v*/*v*, of 20× concentrated solution), lyophilized, and stored at −80 °C until LC-MS analysis. Before LC-MS analysis, the samples were thawed in ice and redissolved in 0.1% FA solution.

### 3.3. LC-MS Peptidomic Analysis

LC-ESI-MS/MS analyses were performed in triplicate on an UltiMate 3000 RSLCnano System coupled to an Orbitrap Elite MS detector with an EASY-Spray nanoESI source (Thermo Fisher Scientific, Waltham, MA, USA) and Thermo Xcalibur 2.2 computer program (Thermo Fisher Scientific) for instrumental operation and data acquisition. Chromatographic separation was performed on an EASY-Spray PepMap C18 column (15 cm in length × 50 μm of internal diameter (ID), 2 μm particles, 100 Å pore size) (Thermo Fisher Scientific) in coupling with an Acclaim PepMap100 nano-trap cartridge (C18, 5 μm, 100 Å, 300 μm i.d. × 5 mm) (Thermo Fisher Scientific). Separation was performed at 40 °C in gradient elution, at a mobile phase flow rate of 0.3 μL/min, using the aqueous FA solution (0.1%, *v*/*v*) as eluent A and ACN/FA solution (99.9:0.1, *v*/*v*) as eluent B as follows: (i) 5% B (7 min), (ii) from 5% to 35% B (113 min), (iii) from 35% B to 99% (2 min), (iv) 99% B (3 min), (v) from 99% to 1.6% B (2 min), (vi) 1.6% B (3 min), (vii) from 1.6% to 78% B (3 min), (viii) 78% B (3 min), (ix) from 78% to 1.6% B (3 min), (x) 1.6% B (3 min), (xi) from 1.6% to 78% B (3 min), (xii) 78% B (3 min), (xiii) from 78% B to 5% B (2 min), and (xiv) 5% B (20 min). The injection volume was 5 μL. The Orbitrap Elite instrument was operated in positive ionization mode at a 60,000 full scan resolution in a 350–2000 *m*/*z* acquisition range, performing MS/MS fragmentation by collision-induced dissociation (CID, 35% normalized collision energy) of the 20 most intense signals of each MS spectrum in Data-Dependent Scan (DDS) mode. The minimum signal was set to 500.0, the isolation width to 5 *m*/*z*, and the default charge state to +2. MS/MS spectra acquisition was performed at a resolution of 60,000 and setting isolation width of 5 *m*/*z*.

### 3.4. LC-MS Data Elaboration and Bioinformatics Analysis

LC-MS and MS/MS data were elaborated by the Proteome Discoverer (PD) software (version 1.4.1.14, Thermo Fisher Scientific), based on the SEQUEST HT cluster as the search engine against the Swiss-Prot Homo Sapiens proteome (UniProtKb, Swiss-Prot, homo+sapiens released in February 2022, accessed on 21 April 2022), applying the following spectrum filters: minimum precursor mass 350 Da, maximum precursor mass 10,000 Da, total intensity threshold 0.0, and minimum peak count 1. The signal-to-noise (S/N) threshold was set to 1.5. The enzyme was not set as we analyzed full-length undigested sequences. Precursor mass tolerance was set at 10 ppm and fragment mass tolerance at 0.02 Da, and an average precursor mass of False and average fragment mass of False were used. The set dynamic modifications were methionine oxidation (+15.995 Da) and acetylation (any N-Terminus, +42.011 Da). Protein and peptide identifications were validated by the Percolator node, by setting the strict target value of the False Discovery Rate (FDR) at 0.01 and the relaxed value at 0.05. LC-MS data in triplicate per sample pool were analyzed as a multireport file by the PD software. The resulting data were filtered for identification in high confidence of rank 1 peptides. Additional data filtering selected the most reliable data, exclusively considering the unique peptides of a protein identified with high confidence and repeatability in all the triplicate analytical runs (Supplementary Tables S1 and S2). Grouping analyses were performed by the Venny 2.1.0 tool (https://bioinfogp.cnb.csic.es/tools/venny, accessed on 25 March 2025) [85]. The Human Protein Atlas (HPA) database [86,87,88] (https://www.proteinatlas.org, accessed on 11 June 2024) was of reference for peptides’ precursor proteins’ classification inside the specific classes of “cancer-related genes”, “cancer-related genes in Brain”, “candidate cancer biomarkers”, “genes highly expressed in glioma”, “unfavorable prognostic genes in GBM”, “favorable prognostic genes in GBM”, “genes only detected in glioma”, “genes highly expressed in brain” and “genes only detected in brain”. The FindPept tool (https://web.expasy.org/findpept/, accessed on 24 March 2025) and UniProt database (https://www.uniprot.org/, accessed on 26 February 2025) have been utilized for peptide identification and analysis of protein sequences. The MEROPS database (https://www.ebi.ac.uk/merops/, accessed on 21 January 2025) [17] was consulted to investigate on the proteolytic enzymes potentially involved in the cleavage of the specific bonds generating the peptides identified in the present investigation. Relative quantitative analysis of the peptides between the different sample pools used the peptide areas as resulting from the multireport data elaboration by the PD software of the LC-MS raw data, using a label-free approach. Statistical significance in the quantitative variations observed was determined by the non-parametric Student’s *t*-test and one-way ANOVA with Tukey’s Multiple Comparison post-hoc test through the GraphPad PRISM 6.0 software for Windows (GraphPad Software, Boston, MA, USA, www.graphpad.com, accessed on 17 February 2025). Protein–protein functional interaction analysis was performed by STRING (version 12.0, accessed on 5 June 2025) [89] with Cytoscape (version 3.10.3) visualization. STRING statistical significance is defined by the FDR with Benjamini–Hochberg correction [90].

## 4. Conclusions

This study illustrates a preliminary overview of the LC-MS proteomic profile of the low molecular mass fraction of the proteome of ND and R GBM CUSA aspirate fluids collected from the tumor CORE zones and the surrounding periphery, obtained following the top-down approach. The CUSA aspirate fluid represents an innovative biological matrix to investigate, which could have interesting applications in mass spectrometry platforms in the perspective of intraoperative clinical applications. The analysis of the proteome fraction < 10 kDa provided valuable insights into the peptidome of the CUSA fluids of ND and R GBMs and their variation in the diverse zones of collection, i.e., the tumor CORE and periphery and the proximate tissue.

To contextualize our findings, we conducted a comparative analysis with previously published data by integrating information from various sources. These included the Human Protein Atlas, the SPENCER database, and supplementary materials associated with relevant scientific publications [41,46,51,52]. The integration of these resources enabled us to assess the consistency and robustness of our results within the framework of the existing literature. Interestingly, some identified peptides were fragments of precursor proteins classified as cancer-related or highly expressed in GBM, and have been previously identified in GBM CUSA fluid following a bottom-up proteomic approach in our previous investigation; thus, their identification could be consistent with the tumor occurrence and molecular processes involved. Beside the classification of the precursor proteins, the identified peptides have to be considered separate entities which can exhibit a biological activity or not, which is known or still unknown, mirroring that of the parent protein or not, or they could simply represent the phenotype resulting from the activity of specific proteases expressed or over-expressed in the pathological process. Therefore, investigating the peptidome can provide interesting hints on biomarker discovery as well as on the molecular processes involved in tumor onset and progression and, additionally, on the identification of tumor antigen peptides of interest for immunopeptidomics studies and applications. Peptidomic analysis provided insights into biomarker discovery, tumor biology, and the identification of tumor-associated antigen peptides relevant to immunopeptidomics. In this context, the GFAP fragment 375–405 and the AQP4 C-terminal fragment 312–323, both classified as GBM-specific antigen peptides [46], were consistently detected across CUSA samples of all zones. The VIM C-terminal fragment 446–466, found in the ND CORE and peripheral regions, and the R CORE GBM, has previously been identified in medulloblastoma [51], traumatic brain injury [52], and ovarian cancer serum samples, and it is catalogued in the SPENCER database [43], supporting its potential relevance in immunopeptidomics. Similarly, the GFAP fragment 388–405, detected in multiple GBM zones, has been associated with pediatric brain tumors [41]. Notably, the RPL29 and MBP fragment peptides 135–149 and 168–177, respectively, are listed in SPENCER as tumor-specific for various cancers [55,56,57], suggesting their potential as novel neoantigens for immunotherapeutic strategies.

Only for few identified peptides was it possible to predict the potential protease responsible for the related cleavage of the parent protein, using the database of reference.

Special attention was attracted by the peptide fragments peculiar to the tumor periphery and the surrounding tissue, with potential interesting clinical applications, as in intraoperative mass spectrometry-guided surgery decision making, providing an interesting perspective for precision medicine.

The use of CUSA fluid pooled samples in this investigation minimized inter-individual variability for a preliminary overview of the low molecular mass fraction of the proteome that complements previous proteomic data obtained on the same samples with a bottom-up approach [13], and adds new interesting suggestions. Although it is not possible to draw definitive conclusions from the analysis of pooled samples from a limited number of patients, the observation of peptidomic profiles peculiar to the GBM tumor type and different collection zones makes the obtained results intriguing for further investigations and reinforces the role of low molecular mass proteome investigation in cancer biomarker discovery. Although the biological role and origin of specific protein fragments are still far from being fully deciphered and understood, these data undoubtedly highlight the importance of peptidomics in deepening the knowledge of the molecular profile of GBM tumors and in the potential development of new clinical applications.

## Figures and Tables

**Table 1:** List of the 40 peptide sequences, derived from 32 precursor proteins, exclusively identified in the ND GBM CUSA CORE with respect to the R GBM CUSA CORE. Their eventual distribution in the other peripheral zones analyzed is also reported.

Peptide Sequence ^§^	Gene Name	Uniprot Accession *	*m*/*z* (MH^+^) Monoisotopic	PTM	Sequence Position (N- or C-Terminal)	A+	A−
DAHKSEVAHRFKDLGEENFKALVL	ALB	P02768	2753.444	-	24–47	-	-
VSVSEHTW	ANK1	P16157	944.449	-	1755–1763	-	-
VELGTQPATQ	APOA2	P02652	1043.540	-	91–100 (C-Term)	-	-
MVDNWRPAQPLKNRQIKASFK	CA2	P00918	2543.372	M1 (Oxid.)	240–260 (C-Term)	-	-
IEGVDAEDGHGPGEQQ	CFB	P00751	1637.710	-	242–257	-	-
ITREEKPAVTAAPKK	CRYAB	P02511	1638.953	-	161–175 (C-Term)	-	-
FDQFFGEHL	CRYAB	P02511	1139.518	-	24–32	-	-
DVELLKLE	FKBP1A	P62942	958.547	-	101–108 (C-Term)	-	-
ESARQDEGKEEEPEADQEHA	GAP43	P17677	2283.961	-	219–238 (C-Term)	-	-
PDPAKSAPAP	H2BC17	P23527	950.496	-	2–11 (N-Term)	-	-
GGGYDNYGGGNY	HNRNPA2B1	P22626	1193.454	-	284–295	-	-
AAGVEAAAEVAATEIK	HNRNPM	P52272	1542.807	Acetyl (N-Term)	2–17 (N-Term)	-	-
SDEFSLADALPEHSPAKTSAVS	MAPK1IP1L	Q8NDC0	2301.099	Acetyl (N-Term)	2–23 (N-Term)	-	-
EDMIRLGDLNEAGILR	MYO7A	Q13402	1814.945	-	68–83	-	-
LEGKVLPGVDALSNI	PGK1	P00558	1524.867	-	403–417 (C-Term)	-	-
AANFLLQQNFDED	RAD23B	P54727	1524.701	-	397–409 (C-Term)	-	-
KAPAQKVPAQKATGQKAAPAPKA	RPL14	P50914	2257.317	-	173–194	-	-
MEDLDQSPLVSSSDSPPRPQPAFKY	RTN4	Q9NQC3	2833.341	Acetyl (N-Term)	1–25 (N-Term)	-	-
KTIAYGYIHDPSGGPVSLDFVKSGDYALERM	SARDH	Q9UL12	3402.681	M_31_(Oxi)	863–893	-	-
LVRYTKKVPQVSTPTL	ALB	P02768	1830.091	-	432–447	x	-
GEYKFQNALL	ALB	P02768	1182.618	-	423–432	x	-
AEYGTLLQDLTNN	PEA15	Q15121	1493.716	Acetyl (N-Term)	2–14 (N-Term)	x	-
EGKVLPGVDALSNI	PGK1	P00558	1411.782	-	404–417 (C-Term)	x	-
AASGAEPQVLVQY	PTRHD1	Q6GMV3	1374.695	Acetyl (N-Term)	16–28	x	-
VDIINAKQ	TPI1	P60174	900.518	-	242–249 (C-Term)	x	-
SPTVSQVTERSQD	ANK1	P16157	1433.692	-	1686–1698	x	x
SSKITHRIHWESASLLR	C3	P01024	2021.109	-	1304–1320	x	x
MVDNWRPAQPLKNRQIKASFK	CA2	P00918	2527.375	-	240–260 (C-Term)	x	x
SSEPPPPPQPPTHQA	CARHSP1	Q9Y2V2	1608.773	Acetyl (N-Term)	2–16 (N-Term)	x	x
SARQDEGKEEEPEADQEHA	GAP43	P17677	2154.921	-	220–238 (C-Term)	x	x
SETPDQFMTADETRNLQ	CD44	P16070	1982.878	-	718–734	x	x
SYTLDSLGNPSA	NEFM	P07197	1266.589	Acetyl (N-Term)	2–13 (N-Term)	x	x
FVSNHAY	ALDOA	P04075	837.391	-	358–364 (C-Term)	-	x
FVELGTQPATQ	APOA2	P02652	1190.607	-	90–100 (C-Term)	-	x
FVQMMTAK	CALM3	P0DP25	955.477	-	142–149 (C-Term)	-	x
ASGVAVSDGVIKVF	CFL1	P23528	1390.761	Acetyl (N-Term)	2–15 (N-Term)	-	x
DDIGDDDEDIDDI	EIF1AY	O14602	1464.554	-	132–144 (C-Term)	-	x
AGWNAYIDNL	PFN1	P07737	1178.551	Acetyl (N-Term)	2–11 (N-Term)	-	x
ISHELDSASSEVN	SPP1	P10451	1387.638	-	302–314 (C-Term)	-	x
AERPAEETGPQEEEGETAGEAPVSH	SPTB	P11277	2607.157	-	2082–2106	-	x

^§^ Peptide fragments of proteins classified as highly expressed in GBM, associated with poor prognosis in GBM, or cancer-related in the HPA database are underlined. * Underlined Uniprot accessions have been identified in the ND CUSA CORE as exclusive by the bottom-up proteomic approach in our previous investigation [13].

**Table 2:** List of the 132 peptide sequences, derived from 46 precursor proteins, identified as common to the ND and R GBM CUSA CORE zones. Their eventual distribution in the peripheral zones is also reported.

Peptide Sequence ^§^	Gene Name	Uniprot Accession *	*m*/*z* (MH^+^) Monoisotopic	PTM	Sequence Position (N- or C-Terminal)	A+	A−	A+	A−
						ND	ND	R	R
VIKAVDKKAAGAGKVTKSAQKAQKA	EEF1A1	P68104	2496.497	-	2–13 (N-Term)	-	-	-	-
FLAEGGGVR	FGA	P02671	905.487	-	27–35	-	-	-	-
RNIVVKTVEMRDGEVIKE	GFAP	P14136	2115.164	-	406–423	-	-	-	-
SKPHSEAGTAFIQTQQLH	PKM	P14618	2022.009	Acetyl (N-Term)	2–19 (N-Term)	-	-	-	-
KNPLPSKETIEQEKQAGES	TMSB4X	P62328	2113.084	-	26–44 (C-Term)	-	-	-	-
IENEEQEYVQTVK	ANXA1	P04083	1608.778	-	14–26	x	-	-	-
VGVIKAVDKKAAGAGKVTKSA	EEF1A1	P68104	1998.209	-	435–455	x	-	-	-
YDMNAANVGWNNSTFA	MIF	P14174	1790.75	M_3_ (Oxi)	100–115 (C-Term)	x	-	-	-
NAANVGWNNSTFA	MIF	P14174	1365.621	-	103–115 (C-Term)	x	-	-	-
TLPTKETIEQEKRSEIS	TMSB10	P63313	1989.056	-	28–43	x	-	-	-
KTETQEKNPLPSKETIEQEKQAG	TMSB4X	P62328	2613.355	-	20–42	x	-	-	-
LSALEEYTKKLNTQ	APOA1	P02647	1637.879	-	254–267 (C-Term)	-	x	-	-
RETSLDTKSVSEGHL	GFAP	P14136	1658.838	Acetyl (N-Term)	390–404	-	x	-	-
IRETSLDTKSVSE	GFAP	P14136	1464.76	-	389–401	-	x	-	-
ETSLDTKSVSEGHL	GFAP	P14136	1502.737	-	391–404	-	x	-	-
ASVSTVLTSKY	HBA1; HBA2	P69905	1155.627	-	131–141	-	x	-	-
VLSPADKTNVKAAWGKVG	HBA1; HBA2	P69905	1841.035	-	2–19 (N-Term)	-	x	-	-
IEEQKIVVK	RPL34	P49207	1085.659	-	97–105	-	x	-	-
TQEKNPLPSKETIEQEKQAGES	TMSB4X	P62328	2471.235	-	23–44 (C-Term)	-	x	-	-
GKVKVGVNGFGRIG	GAPDH	P04406	1387.82	-	2–15 (N-Term)	x	x	-	-
MRDGEVIKESKQEHKDVM	GFAP	P14136	2159.061	-	415–432 (C-Term)	x	x	-	-
PAAPAPAEKTPVKKKARK	H1-4	P10412	1888.15	-	8–26	x	x	-	-
SETAPAETATPAPVEKSPAK	H1-5	P16401	2024.028	Acetyl (N-Term)	2–21 (N-Term)	x	x	-	-
VSTVLTSKY	HBA1; HBA2	P69905	997.558	-	133–141	x	x	-	-
AEAFDDVVGETVGKTD	MAP4	P27816	1652.77	-	31–46	x	x	-	-
YDMNAANVGWNNSTFA	MIF	P14174	1774.752	-	100–115 (C-Term)	x	x	-	-
AELQEVQITE	NDRG2	Q9UN36	1201.597	Acetyl (N-Term)	2–11 (N-Term)	x	x	-	-
KEEIIKTLSKEEETK	RPL19	P84098	1804.995	-	181–195	x	x	-	-
AQAAAPASVPAQAPK	RPL29	P47914	1377.753	-	135–149	x	x	-	-
ASSDIQVKELEKRASGQAF	STMN1	P16949	2106.087	Acetyl (N-Term)	2–20 (N-Term)	x	x	-	-
SDKPDMAEIEKFDKSKL	TMSB4X	P62328	2023.007	Acetyl (N-Term)	2–18 (N-Term)	x	x	-	-
SDKPDMAEIEKFDKS	TMSB4X	P62328	1781.83	Acetyl (N-Term)	2–16 (N-Term)	x	x	-	-
SDEREVAEAATGEDA	VAT1	Q99536	1591.676	Acetyl (N-Term)	2–16 (N-Term)	x	x	-	-
TVETRDGQVINETSQHHDDLE	VIM	P08670	2423.117	-	446–466 (C-Term)	x	x	-	-
TVETRDGQVINETS	VIM	P08670	1548.756	-	446–459	x	x	-	-
QIRETSLDTKSVSEGHLK	GFAP	P14136	2028.076	-	388–405	-	-	x	x
GKRKASGPPVSELITK	H1-3	P16402	1667.981	-	32–47	-	-	x	x
LASVSTVLTSKYR	HBA1; HBA2	P69905	1424.812	-	130–142 (C-Term)	-	-	x	x
ALLSPYSYSTTAVVTNPKE	TTR	P02766	2041.057	-	129–147 (C-Term)	-	-	x	x
GYTEHQVVSSDFNSDTH	GAPDH	P04406	1922.819	-	275–291	x	-	x	-
ADKPDMGEIASFDKAKLKKTETQEKN	TMSB10	P63313	2980.501	Acetyl (N-Term)	2–27 (N-Term)	x	-	x	-
DSYDSYATHNE	CIRBP	Q14011	1301.497	-	162–172 (C-Term)	x	x	x	-
EGDFLAEGGGVR	FGA	P02671	1206.577	-	24–35	x	x	x	-
RDGEVIKESKQEHKDVM	GFAP	P14136	2028.02	-	416–432 (C-Term)	x	x	x	-
TENSTSAPAAKPK	H1-0	P07305	1343.682	Acetyl (N-Term)	2–14 (N-Term)	x	x	x	-
PAAPAAPAPAEKTPVKKKAR	H1-4	P10412	1999.184	-	6–25	x	x	x	-
GAAKRKASGPPVSELITK	H1-4	P10412	1810.054	-	29–46	x	x	x	-
VLSPADKTNVKAAWGKVGAHAGEYGAEALER	HBA1; HBA2	P69905	3195.662	-	2–32 (N-Term)	x	x	x	-
PAVHASLDKF	HBA1; HBA2	P69905	1084.579	-	120–129	x	x	x	-
HFDLSHGSAQVK	HBA1; HBA2	P69905	1325.662	-	46–57	x	x	x	-
SPKAEDGATPSPSNETPKK	MARCKS	P29966	1940.961	-	135–153	x	x	x	-
SPKAEDGATPSPSNETPK	MARCKS	P29966	1812.866	-	135–152	x	x	x	-
FEGFPDKQPR	S100A4	P26447	1220.606	-	90–99	x	x	x	-
LKKTETQEKNPLPSKETIEQEKQAGES	TMSB4X	P62328	3070.6	-	18–44 (C-Term)	x	x	x	-
TVETRDGQVINETSQ	VIM	P08670	1676.815	-	446–460	x	x	x	-
IRETSLDTKSVSEGHL	GFAP	P14136	1771.925	-	389–404	x	x	-	x
IKTVETRDGQVINETSQHHDDLE	VIM	P08670	2664.295	-	444–466 (C-Term)	x	x	-	x
IKTVETRDGQVINETSQ	VIM	P08670	1917.994	-	444–460	x	x	-	x
EIENPETSDQ	GYPA	P02724	1161.493	-	141–150 (C-Term)	-	x	x	-
SETAPAETATPAPVEKSPAKKKAT	H1-5	P16401	2452.305	Acetyl (N-Term)	2–25 (N-Term)	-	x	x	-
FLSFPTTKTY	HBA1; HBA2	P69905	1204.628	-	34–43	-	x	x	-
AEDVTAALAKQGL	MAP2	P11137	1286.697	-	1815–1827 (C-Term)	-	x	x	-
SEEIITPVY	CAPN1	P07384	1092.547	Acetyl (N-Term)	2–10 (N-Term)	-	x	-	x
APPGGRANITSLG	DPYSL2	Q16555	1210.657	-	560–572 (C-Term)	-	x	-	x
VNDNEEGFFSA	FGB	P02675	1228.514	-	33–43	-	x	-	x
SETAPAAPAAPAPAEKTPVKKK	H1-4	P10412	2202.219	Acetyl (N-Term)	2–23 (N-Term)	-	x	-	x
ENPVVHFF	MBP	P02686	988.491	-	217–224	-	x	-	x
DLEPTVIDEVRTGTY	TUBA1B	P68363	1707.847	-	69–83	-	x	-	x
GEGDFLAEGGGVR	FGA	P02671	1263.596	-	23–35	-	x	x	x
MRDGEVIKESKQEHKDVM	GFAP	P14136	2191.046	M_1_(Oxi) M_18_ (Oxi)	415–432 (C-Term)	-	x	x	x
LNETGDEPFQYKN	GLUL	P15104	1554.71	-	361–373 (C-Term)	-	x	x	x
SVEIENPETSDQ	GYPA	P02724	1347.594	-	139–150 (C-Term)	-	x	x	x
GDPALQDAGDSSRKEYFI	GYPC	P04921	1968.935	-	111–128 (C-Term)	-	x	x	x
SETAPAAPAAAPPAEKAPVKKKAA	H1-2	P16403	2314.285	Acetyl (N-Term)	2–25 (N-Term)	-	x	x	x
ASVSTVLTSKYR	HBA1; HBA2	P69905	1311.731	-	131–142 (C-Term)	-	x	x	x
SVSTVLTSKYR	HBA1; HBA2	P69905	1240.693	-	132–142 (C-Term)	-	x	x	x
VHLTPEEKSAVTAL	HBB	P68871	1494.821	-	2–15 (N-Term)	-	x	x	x
GFKGVDAQGTLS	MBP	P02686	1179.603	-	274–285	-	x	x	x
QDENPVVHFF	MBP	P02686	1231.577	-	215–224	-	x	x	x
SLPQKSHGRTQDENPVVH	MBP	P02686	2029.02	-	205–222	-	x	x	x
DDDIAALVVDNG	ACTB	P60709	1258.584	Acetyl (N-Term)	2–13 (N-Term)	x	x	x	x
DDDIAALVVDNGSG	ACTB	P60709	1402.637	Acetyl (N-Term)	2–15 (N-Term)	x	x	x	x
FIENEEQEYVQTVK	ANXA1	P04083	1755.845	-	13–26	x	x	x	x
SEAEDASLLSF	APOC3	P02656	1168.539	-	21–31 (N-Term)	x	x	x	x
GKDQSGEVLSSV	AQP4	P55087	1205.604	-	312–323 (C-Term)	x	x	x	x
LTTDEYDGHSTYPSHQYQ	BLVRB	P30043	2141.912	-	189–206 (C-Term)	x	x	x	x
SAKTSPAKQQAPPVRNLH	DPYSL2	Q16555	1930.065	-	518–535	x	x	x	x
ADSGEGDFLAEGGGVR	FGA	P02671	1536.695	-	20–35	x	x	x	x
DSGEGDFLAEGGGVR	FGA	P02671	1465.658	-	21–35	x	x	x	x
GDFLAEGGGVR	FGA	P02671	1077.535	-	25–35	x	x	x	x
IRETSLDTKSVSEGHLK	GFAP	P14136	1900.017	-	389–405	x	x	x	x
MRDGEVIKESKQEHKDVM	GFAP	P14136	2175.054	M_18_(Oxi)	415–432 (C-Term)	x	x	x	x
RDGEVIKESKQEHKDVM	GFAP	P14136	2044.012	M_17_(Oxi)	416–432 (C-Term)	x	x	x	x
RNIVVKTVEMRDGEVIKE	GFAP	P14136	2131.157	M_10_(Oxi)	406–423	x	x	x	x
SNLQIRETSLDTKSVSEGHLK	GFAP	P14136	2342.235	-	375–405	x	x	x	x
RNIVVKTVEMRDGEVIKESKQEHKDVM	GFAP	P14136	3229.674	M_10_(Oxi) M_27_ (Oxi)	406–432 (C-Term)	x	x	x	x
SLDTKSVSEGHLK	GFAP	P14136	1400.74	-	393–404	x	x	x	x
SSVEIENPETSDQ	GYPA	P02724	1434.629	-	138–150 (C-Term)	x	x	x	x
VEIENPETSDQ	GYPA	P02724	1260.563	-	140–150 (C-Term)	x	x	x	x
SETAPAAPAAPAPAEKTPVK	H1-4	P10412	1946.028	Acetyl (N-Term)	2–21 (N-Term)	x	x	x	x
AAKRKASGPPVSELITK	H1-4	P10412	1753.035	-	30–46	x	x	x	x
SETAPAAPAAPAPAEKTPVKKKAR	H1-4	P10412	2429.354	Acetyl (N-Term)	2–25 (N-Term)	x	x	x	x
SETAPAAPAAPAPAEKTPVKKKARK	H1-4	P10412	2557.452	Acetyl (N-Term)	2–26 (N-Term)	x	x	x	x
SETAPAAPAAPAPAEKTPVKKKA	H1-4	P10412	2273.258	Acetyl (N-Term)	2–24 (N-Term)	x	x	x	x
SETAPAAPAAPAPAEKTPVKK	H1-4	P10412	2074.126	Acetyl (N-Term)	2–22 (N-Term)	x	x	x	x
VLSPADKTNVKAAWGKVGAHAGEYGAEALERM	HBA1; HBA2	P69905	3342.701	M_32_(Oxi)	2–33 (N-Term)	x	x	x	x
AAHLPAEFTPAVHASLDKF	HBA1; HBA2	P69905	2022.049	-	111–128	x	x	x	x
LASVSTVLTSKY	HBA1; HBA2	P69905	1268.711	-	130–141	x	x	x	x
FDLSHGSAQVK	HBA1; HBA2	P69905	1188.604	-	47–57	x	x	x	x
GKVNVDEVGGEALG	HBB	P68871	1343.684	-	17–30	x	x	x	x
VHLTPEEKSAVT	HBB	P68871	1310.696	-	2–13 (N-Term)	x	x	x	x
LSAKPAPPKPEPKPK	HMGN2	P05204	1584.946	-	28–42	x	x	x	x
SARLSAKPAPPKPEPKPK	HMGN2, HMG17	P05204	1899.117	-	25–42	x	x	x	x
SARLSAKPAPPKPEPKPR	HMGN3	Q15651	1927.124	-	28–44	x	x	x	x
TEKSNIEETTISSQKI	INA	Q16352	1807.93	-	484–499 (C-Term)	x	x	x	x
YGSLPQKSHGRTQDENPVVH	MBP	P02686	2249.113	-	203–222	x	x	x	x
TQDENPVVHFF	MBP	P02686	1332.623	-	214–224	x	x	x	x
DTGILDSIGR	MBP	P02686	1046.55	-	168–177	x	x	x	x
DTGILDSIGRF	MBP	P02686	1193.619	-	168–178	x	x	x	x
GRTQDENPVVHF	MBP	P02686	1398.678	-	212–223	x	x	x	x
GSLPQKSHGRTQDENPVVH	MBP	P02686	2086.043	-	204–222	x	x	x	x
FLLSQNFDDE	RAD23A	P54725	1227.553	-	354–363 (C-Term)	x	x	x	x
KTETQEKNPLPSKETIEQEKQ	TMSB4X	P62328	2485.284	-	20–40	x	x	x	x
KTETQEKNPLPSKETIEQEKQAGES	TMSB4X	P62328	2829.421	-	20–44 (C-Term)	x	x	x	x
SDKPDMAEIEKFDKS	TMSB4X	P62328	1797.826	Acetyl (N-Term)	2–16 (N-Term)	x	x	x	x
SDKPDMAEIEKFDKSK	TMSB4X	P62328	1925.919	Acetyl (N-Term)	2–17 (N-Term)	x	x	x	x
LLSPYSYSTTAVVTNPKE	TTR	P02766	1970.017	-	130–147 (C-Term)	x	x	x	x
VDLEPTVIDEVR	TUBA1B	P68363	1384.735	-	68–79	x	x	x	x
DLEPTVIDEVR	TUBA1B	P68363	1285.667	-	69–79	x	x	x	x
LIKTVETRDGQVINETSQHHDDLE	VIM	P08670	2777.378	-	443–466 (C-Term)	x	x	x	x
RDGQVINETSQHHDDLE	VIM	P08670	1992.905	-	450–466 (C-Term)	x	x	x	x
LIKTVETRDGQVINETSQ	VIM	P08670	2031.078	-	443–460	x	x	x	x

^§^ The peptide sequences from precursor proteins classified as highly expressed in GBM, associated with poor prognosis in GBM, and cancer-related in the Human Protein Atlas database are underlined. * Underlined Uniprot accession numbers have been identified in both the ND and R CUSA CORE as exclusive by the bottom-up proteomic approach in our previous investigation [13].

**Table 3:** List of the 24 peptide sequences, derived from 18 precursor proteins, identified as exclusive in R GBM CORE CUSA fluid. Their eventual distribution in the peripheral zones is also reported.

Peptide Sequence ^§^	Gene Name	Uniprot Accession *	*m*/*z* (MH^+^) Monoisotopic	PTM	Sequence Position (N- or C-Terminal)	A+	A−
FSNKITPIQSKEAY	AK4	P27144	1625.859	-	210–223 (C-Term)	-	-
GELAKHAVSEGTKAVTK	H2BK1	A0A2R8Y619	1725.954	-	101–117	-	-
RDNIQGITKPAIRR	H4C1, H4C2, H4C3, H4C4, H4C5	P62805	1637.960	-	24–37	-	-
FRDGDILGKYVD	HSPE1	P61604	1397.711	-	91–102 (C-Term)	-	-
ATLKEKLIAPVA	LDHB	P07195	1295.797	Acetyl (N-Term)	2–13 (N-Term)	-	-
LEEENQESLR	NES	P48681	1246.596	-	882–893	-	-
GPGPGGPGGAGVARGGAGGGP	NRGN	Q92686	1559.774	-	55–75	-	-
ASGNARIGKPAPDFK	PRDX2	P32119	1570.839	Acetyl (N-Term)	2–16 (N-Term)	-	-
IVKPVKVSAPRVGGK	RPL24	P83731	1534.981	-	142–156	-	-
IAKLEKAKAKELATKLG	RPL7A	P62424	1812.138	-	250–266 (C-Term)	-	-
SQDEVKAETIRSL	SYN1	P17600	1475.774	-	682–695	-	-
ANEDEAKAETIRSL	SYN2	Q92777	1546.775	-	559–572	-	-
TEPLPEKTQESL	TPD52	P55327	1371.706	-	212–224 (C-Term)	-	-
SPKVSDTVVEPYN	TUBB	P07437	1434.715	-	172–184	-	-
SHEKSFLVSGDN	GRINA	Q7Z429	1361.637	Acetyl (N-Term)	2–13 (N-Term)	x	-
KLEKEEEEGISQESSEEEQ	HMGA1	P17096	2237.003	-	89–107 (C-Term)	x	-
VELQKEEAQKL	HNRNPU	Q00839	1314.729	-	655–665	x	-
GPGGPGGAGVARGGAGGGP	NRGN	Q92686	1405.702	-	57–75	x	-
DFGSLSNLQVTQP	RPS17	P08708	1405.697	-	110–122	x	-
EIVHIQAGQCG	TUBB	P07437	1154.566	-	3–13	x	-
GGPGGAGVARGGAGGGPSGD	NRGN	Q92686	1510.707	-	59–78 (C-Term)	x	x
RKGPGPGGPGGAGVARGGAGGGPSGD	NRGN	Q92686	2103.056	-	53–78 (C-Term)	x	x
GGPGGAGVARGGAGGGP	NRGN	Q92686	1251.623	-	59–75	x	x
RTGPPTTQQPRPSGPGPAGRPKP	SYN1	P17600	2.337.261	-	612–634	-	x

^§^ The peptides with precursor proteins classified as highly expressed in GBM, associated with poor prognosis in GBM, and cancer-related in the Human Protein Atlas database are underlined. * Underlined Uniprot accession numbers have been previously identified in the R CUSA CORE as exclusive by the bottom-up proteomic approach in our previous investigation [13].

**Table 4:** List of the 13 peptide sequences (seven precursor proteins) identified as exclusive of ND or R GBM CUSA of the A+ zones.

Peptide Sequence ^§^	Gene Name	Uniprot Accession	*m*/*z* (MH^+^) Monoisotopic	PTM	Sequence Position (N- or C-terminal)	A+ ND	A + R
VDLEPTVIDEVRTGTY	TUBA1C	Q9BQE3	1806.915	-	68–83	x	-
SDKTIGGGDDSFNT	TUBA1C	Q9BQE3	1413.618	-	38–51	x	-
EQLITGKEDAANNY	TUBA1C	Q9BQE3	1565.749	-	90–103	x	-
VDLEPTVIDEVR	TUBA1C	Q9BQE3	1384.737	-	68–79	x	-
DLEPTVIDEVR	TUBA1C	Q9BQE3	1285.667	-	69–79	x	-
SETGAGKHVPRAVF	TUBA1C	Q9BQE3	1455.774	-	54–67	x	-
DLTEFQTNL	TUBA1C	Q9BQE3	1080.521	-	251–259	x	-
KVEQAVETEPEPELRQQTE	APOE	P02649	2240.111	-	19–37 (N-term)	x	-
AATDLERFSNAEPEPR	SEPTIN8	Q92599	1844.881	Acetyl (N-Term)	2–17 (N-term)	x	-
ATDTSQGELVHPK	HP1BP3	Q5SSJ5	1424.707	Acetyl (N-Term)	2–14 (N-term)	x	-
SARLSAKPAPPKPEPRPKKASA	HMGN4	O00479	2284.328	-	25–46	x	-
EIRRYQKSTELLIR	H3C1	P68431	1805.045	-	51–64	-	x
YIAALGGAGGKGNRFFLAN	MTG2	Q9H4K7	1897.015	-	180–198	-	x

^§^ The peptides with precursor proteins classified as highly expressed in GBM, associated with poor prognosis in GBM, and cancer-related in the Human Protein Atlas database are underlined.

**Table 5:** List of the 18 peptide sequences, derived from 14 precursor proteins, identified as exclusive in the ND or R GBM CUSA fluids of the A− zones.

Peptide Sequence ^§^	Gene Name	Uniprot Accession	*m*/*z* (MH^+^) Monoisotopic	PTM	Sequence Position (N- or C-Terminal)	A− ND	A− R
ADPGSDLFSVQALQ	SPTA1	P02549	1447.712	-	1209–1222	x	-
MVEEDEHEPKFEKF	SPTA1	P02549	1793.809	-	2309–2322	x	-
DPGSDLFSVQALQ	SPTA1	P02549	1376.675	-	1210–1222	x	-
TAEEIQERRQEVLT	SPTA1	P02549	1701.884	-	20–33	x	-
AQSKGNPEQTPVLKPEEEAPAPEVG	VTN	P04004	2602.311	-	104–128	x	-
RDQKLSELDDRADALQ	VAMP3	Q15836	1872.948	-	39–54	x	-
EEEIAALVIDNG	ACTG1	P63261	1314.644	Acetyl (N-Term)	2–13 (N-term)	x	-
AVGGEALGRL	HBD	P02042	942.539	-	23–32	x	-
AEDMETKIKNY	COX6B1	P14854	1383.653	Acetyl (N-Term)	2–12 (N-term)	x	-
SIQENISSL	PKLR	P30613	1032.522	Acetyl (N-Term)	2–10 (N-term)	x	-
TVATPLNQVANPNSAIFGGARPREEVVQKEQE	EIF4H	Q15056	3449.790	-	217–248 (C-term)	x	-
IEDVTPIPSDSTRRKGGRRGR	RPS14	P62263	2353.286	-	129–149	x	-
EEPAAHDTEATATDYHTT	SLC4A1	P02730	1959.828	-	32–49	x	-
ASTEGANNMPKQVEVRMHDSHLG	SLC1A2	P43004	2550.182	Acetyl (N-Term)	2–24 (N-term)	x	-
WEVISDEHGIDPTGS	TUBB2A	Q13885	1641.745	-	21–35	x	-
ADEVSASLAKQGL	MAPT	P10636	1288.678	-	746–758 (C-term)	x	-
TLADEVSASLAKQGL	MAPT	P10636	1502.813	-	744–758 (C-term)	x	-
EIRRYQKSTELLIR	H3-5	Q6NXT2	1805.048	-	50–63	-	x

^§^ The peptides with precursor proteins classified as highly expressed in GBM, associated with poor prognosis in GBM, and cancer-related in the Human Protein Atlas database are underlined.

## Data Availability

The mass spectrometry proteomics data have been deposited at the ProteomeXchange Consortium via the PRIDE [91] partner repository, with the dataset identifier PXD060807.

## Supplementary Materials

The following supporting information can be downloaded at: https://www.mdpi.com/article/10.3390/ijms26136055/s1.

## Abbreviations

The following abbreviations are used in this manuscript:

GBM	Glioblastoma multiforme
ND GBM	Newly diagnosed GBM
R GBM	Recurrent GBM
CUSA	Cavitron Ultrasonic Surgical Aspirator
FASP	Filter-Aided Sample Preparation
LC	Liquid Chromatography
MS	Mass spectrometry
CNS	Central nervous system
FA	Formic acid
5-ALA	5-aminolevulinic acid
ACN	acetonitrile
PIC	Protease inhibitor cocktail
FDR	False Discovery Rate
HPA	Human Protein Atlas
