# Supplementary material for: Proteomic Analysis of the Low Molecular Mass Fraction of Newly Diagnosed and Recurrent Glioblastoma CUSA Fluid: A Pilot Investigation of the Peptidomic Profile
Source: Int J Mol Sci. 2025 Jun 24;26(13):6055. doi: 10.3390/ijms26136055 (PMC12249807; doi:10.3390/ijms26136055)
Supplement: Supplementary file 1 [file ijms-26-06055-s001.zip › TableS3.pdf]

Manuscript: Proteomic analysis of the low molecular mass fraction of newly diagnosed and recurrent glioblastoma CUSA fluid: a pilot investigation of the peptidomic profile

Table S3. Patient data summary.

Patient ID	age (year)	gender (M=0; F=1)	GBM ND=newly diagnosed R=relapse	lobe of the lesion (frontal =0; temporal =1; parietal =2; occipital =3)	lesion side (left=0; right=1)	IDH-1 (wt=0;mutated=1)
**PP1**	57	0	ND	2/3	0	0
**PP2**	73	0	ND	2	0	0
**PP3**	52	0	ND	2	0	0
**PP4**	50	0	R	1	0	0
**PP5**	56	0	R	2	1	0
**PP6**	40	1	R	2	1	0
**PP7**	75	0	ND	2/3	1	0
